# Effectiveness and Tolerability of the Intensification of Canagliflozin Dose from 100 mg to 300 mg Daily in Patients with Type 2 Diabetes in Real Life: The INTENSIFY Study

**DOI:** 10.3390/jcm12134248

**Published:** 2023-06-25

**Authors:** Juan J. Gorgojo-Martinez, Pablo José Ferreira-Ocampo, Alba Galdón Sanz-Pastor, Jersy Cárdenas-Salas, Teresa Antón-Bravo, Miguel Brito-Sanfiel, Francisca Almodóvar-Ruiz

**Affiliations:** 1 Department of Endocrinology and Nutrition, Hospital Universitario Fundación Alcorcón, 28922 Madrid, Spain; 2 Department of Endocrinology and Nutrition, Hospital Universitario Gregorio Marañón, 28007 Madrid, Spain; albagaldonsp@gmail.com; 3 Department of Endocrinology and Nutrition, Fundación Jiménez Díaz, 28040 Madrid, Spain; 4 Department of Endocrinology and Nutrition, Hospital Universitario de Móstoles, 28935 Madrid, Spain; tantonb1978@gmail.com; 5 Department of Endocrinology and Nutrition, Hospital Universitario Puerta de Hierro, 28222 Madrid, Spain; mbritosanfiel@gmail.com

**Keywords:** SGLT-2 inhibitor, canagliflozin, type 2 diabetes, switch, intensification

## Abstract

**Aim:** This study aimed to evaluate the effectiveness and tolerability of intensifying the dose of canagliflozin from 100 mg/day (CANA100) to 300 mg/day (CANA300) in patients with type 2 diabetes (T2DM) and suboptimal metabolic control in a real-world setting. **Methods:** A multicenter observational study was conducted on adult patients with T2DM who initiated treatment with CANA100 and subsequently required intensification to CANA300. The primary outcome measures were changes in HbA1c and weight at 6 months after the switch and at the end of the follow-up period. **Results:** A total of 317 patients met the inclusion criteria (59.6% male, mean age 62.2 years, baseline HbA1c 7.55%, weight 88.6 kg, median duration of treatment with CANA100 9.9 months). Switching to CANA300 resulted in a significant reduction in HbA1c (6 months: −0.33%; last visit: −0.47%, both *p* < 0.0001) and weight (6 months: −1.8 kg; last visit: −2.9 kg, both *p* < 0.0001) over a median follow-up period of 20.8 months. The proportion of patients that achieved HbA1c < 7% increased from 26.7% with CANA100 to 51.6% with CANA300 (*p* < 0.0001). Among individuals with poor baseline glycemic control (HbA1c > 8%, mean 9.0%), HbA1c was significantly reduced by −1.24% (*p* < 0.0001). Furthermore, significant improvements were observed in fasting plasma glucose (FPG), blood pressure (BP), liver enzymes, and albuminuria. No unexpected adverse events were reported. **Conclusions**: Intensifying the treatment to CANA300 in a real-world setting resulted in further significant and clinically relevant reductions in FPG, HbA1c, weight, and BP in patients with T2DM. The switch was particularly effective in patients with higher baseline HbA1c levels.

## 1. Introduction

Sodium-glucose cotransporter type 2 (SGLT-2) inhibitors are a class of orally administered glucose-lowering drugs (GLDs) that increase urinary glucose excretion (UGE), which induces significant reductions in pre- and postprandial glycemia, HbA1c, body weight, and blood pressure (BP) in patients with type 2 diabetes (T2DM) [1,2]. Clinical trials have also demonstrated cardiovascular (CV) and renal benefits of several SGLT-2 inhibitors in patients with T2DM and high CV risk, heart failure (HF), or chronic kidney disease (CKD) [1]. Moreover, emerging evidence suggests that these benefits extend to non-T2DM patients with HF (the EMPEROR-PRESERVED, EMPEROR REDUCED, DAPA-HF, and DELIVER trials) [3,4] or CKD (the DAPA-CKD and EMPA-KIDNEY trials) [3,5].

In Europe, the SGLT-2 inhibitors (SGLT-2is) currently available are dapagliflozin, empagliflozin, canagliflozin, and ertugliflozin. These drugs induce a UGE ranging from 60 to 80 g per day, except for the higher dose of canagliflozin (300 mg daily), which achieves a UGE of up to 119 g/day [6]. Notably, canagliflozin 300 mg (CANA300) exhibits a unique characteristic among SGLT2-is: it transiently inhibits the sodium-glucose cotransporter type 1 (SGLT-1) in the small intestine, leading to reduced postprandial blood glucose levels [7]. This effect has been observed with doses above 200 mg of canagliflozin in both healthy volunteers and patients with T2DM and is limited to the meal following drug administration [7,8,9]. In a clinical trial involving healthy volunteers, CANA300 demonstrated greater UGE and less postprandial glycemic excursion compared to 10 mg of dapagliflozin [10].

Direct comparisons between 100 mg of canagliflozin daily (CANA100) and CANA300 from phase 3 randomized clinical trials (RCTs) [11,12], as well as indirect comparisons of CANA300 with other SGLT-2is [13,14], have shown that CANA300 induces significantly greater reductions in HbA1c, weight, and systolic blood pressure (SBP) than its comparators. However, although CANA300 seems to show greater efficacy than CANA100, no RCT to date has evaluated the strategy of intensifying SGLT-2i therapy by increasing the dose from CANA100 to CANA300. Existing studies have either compared both arms separately or did not measure the effect of intensification. As a result, it remains unknown whether patients who have an inadequate response to CANA100 will exhibit a decreased response or worse tolerance to CANA300, potentially leading to unnecessary costs.

Real-world evidence from a study conducted in Spain (Real WECAN) supports the possible strategy of intensification with CANA300 in cases of insufficient glycemic control with other SGLT2is [15]. The study showed that patients who switched to CANA300 experienced a significant decrease in HbA1c (−0.35%) and weight (−2.1 kg). Among patients with a baseline HbA1c level greater than 8% (mean 8.94%), the switch to CANA300 resulted in a reduction of the HbA1c levels by 1.12%. The next question to address is whether increasing the canagliflozin dose from 100 mg/day to 300 mg/day in patients with suboptimal metabolic control will achieve similar or greater results compared to the differences observed between the CANA100 and CANA300 arms in RCTs. This strategy, not included in recent guidelines [16], would avoid the need for prescribing new GLDs on top of the current treatment, thereby reducing the treatment burden and complexity. Additionally, the metabolic benefits of CANA300 would complement the cardiovascular and renal protection already demonstrated by CANA100.

The aim of this multicenter RWS study, INTENSIFY, is to assess the effectiveness and tolerability of increasing the canagliflozin dose from 100 mg/day to 300 mg/day in patients with T2DM and suboptimal metabolic control followed in several Departments of Endocrinology and Nutrition in the region of Madrid (Spain).

## 2. Material and Methods

### 2.1. Study Design and Patient Population

We carried out a retrospective multicenter study of a cohort of adult patients with T2DM from five Departments of Endocrinology and Nutrition of university hospitals in the region of Madrid (Spain), who started treatment with CANA100 as part of their antihyperglycemic treatment and who subsequently required an intensification to CANA300 due to insufficient individualized metabolic control. Patients were selected between May 2015 and December 2021 if they fulfilled the following inclusion criteria: (1) individuals older than 18 years with T2DM who started treatment with CANA100 as their first SGLT2i (either 100 mg once daily or 50 mg of canagliflozin in combination with metformin twice daily) with a subsequent escalation to CANA300 (either 300 mg once daily or 150 mg in combination with metformin twice daily), (2) a time between the intensification to CANA300 and enrollment in the study of at least 6 months, (3) an estimated glomerular filtration rate (eGFR) ≥ 60 mL/min/1.73 m^2^, according to the European Medicines Agency label, and (4) the administration of at least one dose of CANA300. The study exclusion criteria were: (1) patients who did not meet the inclusion criteria above; (2) patients previously treated with another SGLT-2i; (3) patients who completed 6 months of CANA300 without data from HbA1c or weight at the initial or final visit; (4) patients who discontinued treatment whose baseline HbA1c and weight data were not available or the reason for discontinuation was not registered on the medical record; and (5) patients with uncontrolled medical illnesses or with pharmacological treatment that might induce a deterioration in glycemic control (corticosteroids, immunosuppressants, somatostatin analogs, etc.).

### 2.2. Outcomes and Study Measures

Patients switching from CANA100 to CANA300 were identified in the diabetes clinic databases, and demographic, clinical, and laboratory variables of interest were collected anonymously from the hospital records. Adverse effects, CV events, drug pauses, and withdrawals (WDs) were also recorded. Participants who interrupted the drug at least once for >7 days but restarted it within the follow-up period were allocated to the interrupters group. Four follow-up visits were defined during the CANA300 treatment period: V1 (switch from CANA100 to CANA300), V2 (6 ± 2 months), V3 (12 ± 2 months), and V4 (final visit, last observation). The following exposure variables were collected: (1) *baseline demographic and clinical variables*: age; gender; ethnic group; weight; height; BMI; SBP; diastolic BP (DBP); heart rate (HR); canagliflozin dosage; background GLDs; hypertension, hypercholesterolemia, and /or hypertriglyceridemia; tobacco use; alcohol intake; sleep apnea; nonalcoholic fatty liver disease (NAFLD); microvascular complications (retinopathy, CKD, and neuropathy); macrovascular complications (ischemic heart disease, cerebrovascular disease, and peripheral arterial disease); HF; arrhythmias; and antihypertensive and lipid-lowering drugs and (2) *laboratory variables*: fasting plasma glucose (FPG); HbA1c; hematocrit; platelets; albumin; total cholesterol; HDL-C; LDL-C; triglycerides; serum uric acid; hepatic serum biomarkers (alanine transaminase (ALT), aspartate aminotransferase (AST), and gamma-glutamyl transferase (GGT)); serum creatinine; eGFR using the CKD-EPI equation; and the urinary albumin to creatinine ratio (UACR). The study outcome variables were changes in HbA1c and weight during visits V2 and V4. The exploratory outcome variables included changes in FPG, BP, hematocrit, uric acid, lipids, liver enzymes, eGFR, UACR, and persistence and adherence during the follow-up. Prior changes in HbA1c, weight, BP, hematocrit, uric acid, liver enzymes, and renal function during the treatment with CANA100 before the switch were also analyzed. A multivariate predictive model for HbA1c reduction after switching to CANA300 was constructed using a multiple linear regression analysis.

The most frequent adverse events (AEs) associated with canagliflozin treatment (genital mycotic infections (GMIs), urinary tract infections (UTIs), fractures, volume depletion events, and polycythemia) were collected by the investigators at each visit. CANA300 withdrawals were documented, specifying the withdrawal date and cause (death, AEs, lack of efficacy, loss of follow-up, and others).

### 2.3. Statistical Analysis

The categorical data are presented as percentages. Continuous variables that follow a normal distribution are expressed as the mean (standard deviation (SD)), and those that do not meet the normality criteria are shown as the median (interquartile range (IQR)). Repeated measures tests and McNemar or Cochran tests were performed to analyze changes in the continuous and categorical variables, respectively. The best predictive model for the response to CANA300 was estimated with a multiple linear regression analysis. In order to select potential predictive variables, B coefficients of each independent variable were obtained by introducing them one by one in the regression model. Control variables which association with the response had a *p*-value > 0.10 were excluded from the multivariate analysis. The selection of the best regression equation for predictive purposes was conducted using the Mallows ordering criterion (lowest Cp value), previously building all the possible sub-models by combining the terms of the maximum model. The sample size was estimated using the formula for paired *t*-tests, based on statistical data from a previous observational study [15]. To detect statistically significant differences in HbA1c at the final visit after switching from CANA100 to CANA300, assuming a minimum expected difference of −0.5%, a standard deviation of 2.5, a power of 90%, a two-sided significance level of 0.05, and accounting for a 20% rate of WS or invalid data, a total of 329 patients were required for inclusion in the study. No other secondary outcome variables were considered in the sample size calculations. A statistical analysis was performed using SPSS software version 15.0.1 (IBM Corp., Armonk, NY, USA) by using 2-sided tests and a significance level of 0.05.

The project was approved by the Ethics Committee of the Hospital Universitario Fundación Alcorcón (ethical code 21/157). The study was carried out in accordance with the ethical principles of the Helsinki Declaration for Medical Research involving Human Subjects and the International Guidelines for Ethical Review of Epidemiological Studies. Given the retrospective design of the study under normal clinical practice conditions, which included patients whose treatment started several years before the inclusion, written informed consent was requested for individuals who attended the clinics during the data collection period; for the rest of the patients, exemption was granted.

## 3. Results

### 3.1. Demographic and Baseline Characteristics

Three hundred and eighty-six patients who had switched from CANA100 to CANA300 were screened; sixty-nine of them did not meet the inclusion criteria or had invalid data, so the final *n* was three hundred and seventeen patients. Table 1 and Table 2 show the baseline characteristics of the cohort. Patients had been previously treated with CANA100 over a median time of 9.9 months, reducing their FPG −27.5 mg/dL (*p* < 0.0001), HbA1c −0.78%, (*p* < 0.0001), weight −2.7 kg (*p* < 0.0001), SBP −5.5 mmHg (*p* = 0.002), and DBP −2.1 mmHg (*p* = 0.025).

### 3.2. Analyses of Effectiveness

**Figure 1:**
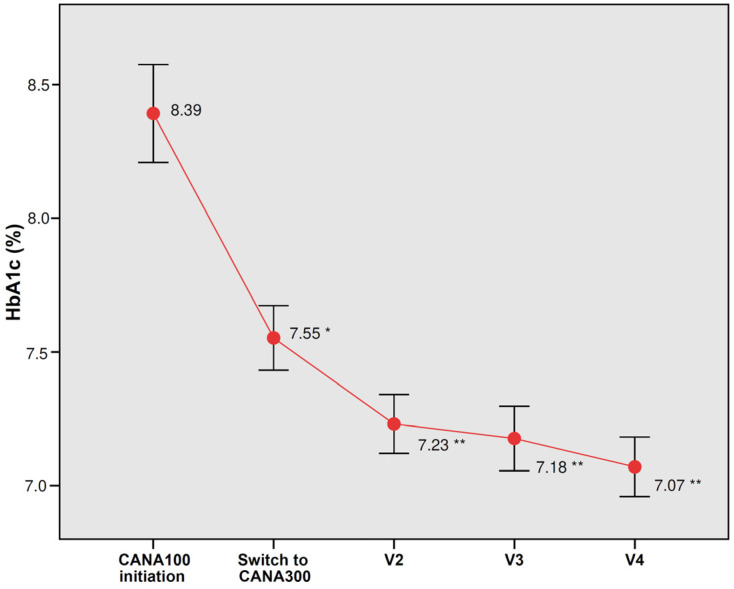
Changes in glycated hemoglobin (HbA1c) with CANA100 (V1) and after switching to CANA300 at 6 months (V2), 12 months (V3), and at the end of the follow-up (V4). Data are the mean (95% confidence interval). * *p* < 0.0001 vs. HbA1c at CANA100 initiation; ** *p* < 0.0001 vs. HbA1c at the switch to CANA300.

The intensification to CANA300 from a baseline HbA1c of 7.55% during a median follow-up of 20.8 months induced a significant decrease in HbA1c −0.47% (*p* < 0.0001); at 6 months, the decrease in HbA1c was −0.33% (*p* < 0.0001) and, at 12 months, −0.40% (*p* < 0.0001) (Figure 1). The percentage of patients with HbA1c below 7% increased significantly from 26.7% with CANA100 to 51.6% with CANA300 (*p* < 0.0001). The percentage of patients with HbA1c below 6.5% increased significantly from 14.5% with CANA100 to 28.3% in the CANA300 treatment period (*p* < 0.0001). In those patients with HbA1c higher than 7% at the switch visit (mean 8.0%), HbA1c was significantly reduced by −0.69% (*p* < 0.0001). In those patients with poor baseline glycemic control (HbA1c above 8%, mean 9.0%), HbA1c was significantly reduced by −1.24% (*p* < 0.0001). In a multivariate multiple linear regression analysis, after analyzing 40 potential predictors, the best predictive model of the HbA1c response at the end of the follow-up included the baseline HbA1c (0.54% better response for each percentage point increase in HbA1c) and treatment with metformin (0.36% worse response in patients treated with metformin), Cp Mallows 2.38, adjusted R^2^ 0.312, *p* < 0.0001 (Table 3). The administration of CANA300 once a day achieved a greater decrease in HbA1c than the administration of CANA150 mg twice a day (−0.65% vs. −0.32%, *p* 0.006). However, after adjusting for other variables, the differences between CANA300 once daily and CANA150 twice daily were not significant. Fewer patients on CANA300 once a day were taking metformin in comparison to those on CANA150 mg twice a day (75.7% vs. 100%, *p* < 0.0001).

**Figure 2:**
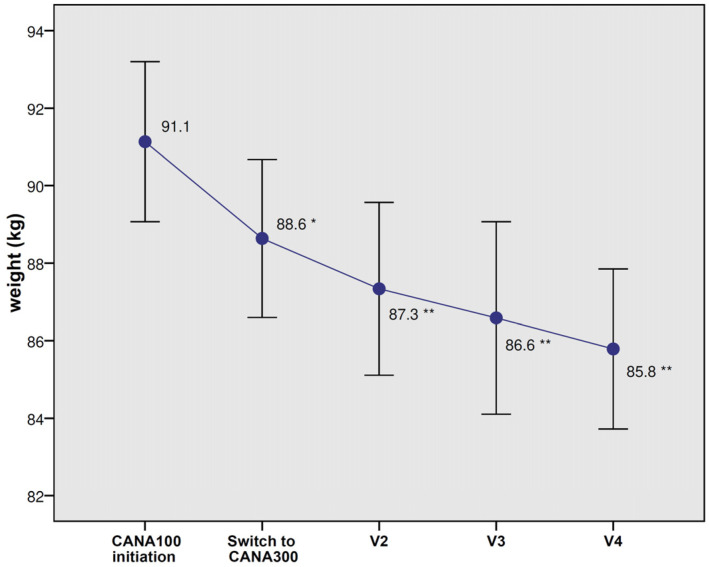
Changes in body weight with CANA100 (V1) and after switching to CANA300 at 6 months (V2), 12 months (V3), and at the end of the follow-up (V4). Data are the mean (95% confidence interval). * *p* < 0.0001 vs. body weight at CANA100 initiation; ** *p* < 0.0001 vs. body weight at switch to CANA300.

A significant weight loss (−2.9 kg, *p* < 0.0001) was observed during the last visit for CANA300 (Figure 2). The weight reductions at 6 and 12 months were −1.8 kg and −2.5 kg, respectively (*p* < 0.0001). A total of 30.1% of patients lost more than 5% of their body weight after the switch to CANA300. Significant improvements in FPG (−14.8 mg/dL, *p* < 0.0001), SBP (−5.3 mmHg, *p* = 0.002), and DBP (−3.1 mmHg, *p* = 0.001) were found at the end of the follow-up (Table 4). In those patients with poorly controlled hypertension (SBP above 140 mmHg, mean 151.2 mmHg), the switch to CANA300 induced a significant decrease in SBP (−17.8 mmHg, *p* < 0.0001). Significant reductions in LDL-C, triglycerides, AST, ALT, and UACR were also observed. In those individuals with a baseline UACR ≥ 30 mg/g, albuminuria was reduced from 166.2 mg/g to 112.9 mg/g (*p* = 0.005). There were no significant changes in eGFR, serum uric acid, or hematocrit; all these variables showed significant changes during the CANA100 period (eGFR −3 mL/min/1.73 m^2^, serum uric acid −0.5 mg/dL, and hematocrit +2,1%, all *p* < 0.05) but leveled off with CANA300.

If we consider the entire treatment period with canagliflozin (initial treatment with CANA100 and later switch to CANA300), with a median follow-up of 38.8 months and baseline HbA1c 8.4%, weight 91.1 kg, SBP 138.9 mmHg, and DBP 79.2 mmHg, patients achieved a statistically significant overall reduction in HbA1c (−1.30%), weight (−5.8 kg), SBP (−9.6 mmHg), and DBP (−4.7 mmHg), all *p* < 0.0001.

There were some changes in the use of other GLDs during the follow-up (Table 5). The percentage of patients on metformin, sulfonylureas, glinides, or insulin remained stable; the use of DPP-4 inhibitors (DPP-4is) decreased; and there was an increase in patients treated with pioglitazone and GLP-1 receptor agonists. In the patients on insulin, the mean dose was reduced from 38.4 U/day to 35.2 U/day. In a sensitivity analysis, the HbA1c reduction in those patients who did not receive GLP-1 receptor agonists during the entire follow-up was very similar to that found in the subgroup of patients on GLP-1 receptor agonists at the baseline and the results observed in the entire cohort (−0.43%, −0.40%, and −0.47%, respectively). There were no relevant changes in the use of antihypertensive drugs, but there was a slight increase in the use of lipid-lowering agents (Table 5).

### 3.3. Analyses of Safety

7.9% of patients discontinued CANA300, the most frequent causes of WDs were GMIs (*n* = 6, 1.9%) and UTIs (*n* = 3, 0.9%). One patient died from sudden death, not attributed to CANA300 by the clinician or the investigator. There was 2.8% of patients with temporary CANA300 interruptions. No statistically significant differences in the posology of CANA300 (150 mg twice daily and 300 mg once daily) were observed between individuals who transiently interrupted the treatment and those who did not interrupt the treatment. During the period of treatment with CANA100, 8.8% of patients experienced GMIs, 4.7% UTIs, and 8.5% non-severe hypoglycemia (23 patients with insulin and 4 patients with sulfonylureas or glinides). Two patients (0.6%) had fractures, and one patient (0.3%) had polyglobulia. There were no events related to volume depletion, episodes of ketoacidosis, or amputations. Over the treatment period with CANA300, 9.5% of patients experienced GMIS, 3.5% UTIs, and 6.0% non-severe hypoglycemia (19 patients with insulin and 1 patient with sulfonylureas or glinides). Four patients (1.3%) had fractures, two patients (0.6%) experienced polyglobulia, and two patients (0.6%) episodes of volume depletion. There were no amputations or ketoacidosis.

## 4. Discussion

The findings from this RWS show that the intensification of canagliflozin therapy in patients with T2DM and suboptimal metabolic control by increasing the dose from 100 to 300 mg daily achieved further significant and clinically relevant reductions in FPG, HbA1c, weight, and BP. The HbA1c reduction observed in those patients with poor glycemic control (−1.24%) was especially noteworthy, since such results would be theoretically expected from the addition of another GLD from a different class. In the multivariate analysis, every one percentage point increase in the baseline HbA1c raised the likelihood of reducing HbA1c by approximately 0.5%. The results from RCTs and other RWS also concluded that a high baseline HbA1c is a predictive factor of the response to SGLT-2is compared to other drugs, such as DPP-4is [17]. This finding might be explained by the mechanism of action of SGLT-2is, as the degree to which UGE is increased in patients taking an SGLT2i is dependent, in part, on the degree of glycemia.

The metabolic benefits observed in the INTENSIFY study resulting from the switch to CANA300 were more pronounced compared to the modest differences observed in RCTs that directly compared CANA100 and CANA300. Nonetheless, it is essential to acknowledge that the retrospective nature of this study inherently offers a lower level of evidence compared to a clinical trial. In a recent meta-analysis of RCTs, the differences between CANA300 and CANA100 were FPG −6.3 mg/dL, HbA1c −0.15%, weight −0.72 kg, SBP −1.1 mmHg, and DBP −0.5 mmHg (all *p* < 0.05) [11]. The differences between low and high doses of other SGLT-2is, including empagliflozin, dapagliflozin, ertugliflozin, luseogliflozin, and ipragliflozin, were even smaller, with little clinical relevance and, in some cases, without statistical significance [11]. Several RWS with canagliflozin have been published in recent years, but none of them explored the switch from CANA100 to CANA300 [18,19,20]. In the observational REAL-WECAN study published by our group, patients who intensified their antihyperglycemic therapy by switching to CANA300 from other SGLT-2is (also including CANA100) showed similar results to those observed in the present study [15]. However, in a recent RWS evaluating the intensification of another SGLT-2i, empagliflozin, from 10 mg to 25 mg, the HbA1c and weight reductions at 24 weeks were only −0.13% and −0.6 kg, respectively [21]. Both RCTs and RWS suggest greater effectiveness of CANA300 within the SGLT-2i class, and intensification to the higher dose of canagliflozin could delay the need to add new drugs and therefore avoid an increase in treatment burden. In fact, sequential treatment with CANA100 and the subsequent change to CANA300 achieved remarkable results in glycemic control (HbA1c −1.30%), weight (−5.8 kg), and BP (−9–6/−4.7 mmHg) in the entire cohort of our study.

The higher efficacy and effectiveness of CANA300 compared to CANA100 and other SGLT-2is are related, on the one hand, to the greater UGE induced by CANA300 as a consequence of a sustained plasma concentration of the drug throughout 24 h and, on the other hand, to the ability to locally inhibit SGLT-1 in the intestine, reducing the postprandial glycemic peak [10]. The administration of CANA300, but not CANA100 or CANA150, delays intestinal glucose absorption, and glucose metabolism by the intestinal microbiome to short-chain fatty acids subsequently stimulates GLP-1 and PYY secretion by L cells [8]. In our study, a greater effect on HbA1c with the administration of CANA300 once daily compared to CANA150 twice a day was observed, although after adjusting for other variables, the differences were not statistically significant. Although metformin was a negative predictor of the glycemic response in the best predictive model, it did not remain significant in a full model, including the canagliflozin dosage. Because more patients taking CANA300 were not also taking metformin compared to patients taking CANA150 twice daily, it is tempting to speculate that the numerical lower effect of canagliflozin seen in patients on metformin might be partially attributed to their different posology. Our study, however, was not designed to find differences between both dosages, so clinical trials specifically powered to answer this question are needed.

Significant decreases in LDL-C and triglycerides were found during the follow-up (Table 4), probably related to a greater use of lipid-lowering agents due to the recommendation for stricter LDL-C control endorsed by the current guidelines.

Increasing the canagliflozin dose in our study was also associated with other parameters, such as liver enzymes or UACR (Table 4). An improvement in liver function tests has been observed in RCTs with canagliflozin and other SLGT-2is and seems to be attributable to weight reduction and better glycemic control [22], which promote fatty acid utilization and the reduction of visceral and liver fat in patients with DM2 and NAFLD [23]. Significant reductions in UACR were observed across the entire cohort, particularly in patients with baseline albuminuria, while eGFR remained unchanged (Table 4). However, it is worth noting that this decline in UACR is likely attributable to multifactorial influences beyond the effects of canagliflozin. Prior treatment with CANA100 had induced an expected drop in eGFR of −3 mL/min/1.73 m^2^ but increasing the dose to 300 mg did not cause a further drop in the filtration rate and renal function remained stable throughout the follow-up. No significant differences between both doses of canagliflozin on eGFR trajectories have been observed in RCTs or RWS [24,25].

The lack of an effect of canagliflozin intensification on the hematocrit and serum uric acid levels is intriguing (Table 4). During the treatment phase with CANA100, there was a significant increase in the hematocrit of 2.1% and a significant reduction in uric acid of −0.5 mg/dL. These parameters did not experience significant changes after the switch to CANA300. The increase in hematocrit is a class effect of SGLT-2i and seems to be a mediator of cardiorenal protective effects, but what causes such an increment is currently unclear. The hemoconcentration seems to be an unlikely explanation after reviewing the current evidence [26,27]. One hypothesis is that the inhibition of glucose and sodium renal reabsorption in the proximal tubule ameliorates cortical hypoxia and metabolic stress and promotes the redifferentiation of myofibroblasts into erythropoietin-producing cells, restores the activity of hypoxia-inducible factor (HIF), increases erythropoietin secretion, and raises the iron utilization by inhibiting hepatic hepcidin production, which ultimately increases hematopoiesis [28]. Other authors have suggested that SGLT-2is upregulate nutrient deprivation signals through sirtuins that activate HIF2α and increase erythropoiesis [29]. In any case, the results from our study suggest that the increase in hematocrit with canagliflozin does not appear to be dose-dependent. Regarding the uric acid levels, SGLT-2is increase urinary the excretion of urate due to the competitive effect of glucose with uric acid on the hexose/urate transporter GLUT-9b in the proximal tubule [30]. Other authors have pointed out the hypothesis of the activation of oxygen and nutrient deprivation signaling as a common link in the increase in hematocrit and the decrease in uric acid, since sirtuins would reduce oxidative stress and increase the tubular secretion of urate [29]. Similar to the effect on hematocrit, the urate-lowering effect of canagliflozin does not appear to be dose-dependent in our cohort of patients either.

The frequency of AEs after intensification to CANA300 was low and without a dose–response relationship. The most common AEs were genitourinary infections, as previously described in RCTs and RWS, but they caused the discontinuation of CANA300 in a small number of patients. It is reassuring to confirm the absence of amputations or ketoacidosis with both doses of canagliflozin over a prolonged period of treatment. The INTENSIFY study had a median follow-up of approximately 21 months, with 43.5% of patients receiving treatment with CANA300 for more than 2 years. The long-term safety of CANA300 has been established in the CANVAS program, a cardiovascular outcome trial that integrated the analyses of two studies, namely CANVAS and CANVAS-R, with a median follow-up of 126 weeks [31]. In the CANVAS trial, 50% of patients were randomized to receive CANA300, while, in the CANVAS-R study, 71.4% of participants in the canagliflozin group had their canagliflozin dosage increased from 100 mg to 300 mg during the trial.

RWS have some limitations. In the present study, a pre-post-evaluation was carried out without a comparison group, and there were some changes in the use of other GLDs, although the sensitivity analyses did not seem to show a relevant influence of these changes on the glycemic outcome. There are possible selection biases that could negatively affect the results. Patients with suboptimal metabolic control after CANA100 were selected, which could include some non-responders, and besides, these patients had been referred to endocrinology departments from university hospitals, which usually attend to more complex patients with a potential worse pharmacological response. However, there might also be a positive selection bias, since patients were followed by expert diabetologists, which could have potentiated the effect of the drug. Finally, it could be argued that, according to our statistical calculation, 329 patients should have been included in the study but the final *n* was 317. However, that sample size was calculated assuming 20% WDs; as only 7.9% of patients discontinued CANA300, the statistical power of the study was maintained above 90%.

In summary, our findings suggest a greater effect of CANA300 compared to CANA100 on various cardiometabolic parameters, as previously demonstrated in RCTs. Additionally, our study provides real-life evidence supporting the switch from CANA100 to CANA300 in patients withT2DM who have suboptimal metabolic control. Notably, the effectiveness of this switch appears to be particularly relevant in individuals with higher HbA1c levels. If confirmed in future switch clinical trials, intensifying the treatment to CANA300 could potentially delay the need for treatment escalation, thereby alleviating the treatment burden.

## Figures and Tables

**Table 1:** Baseline characteristics of the patients switching from CANA100 to CANA300. Data: percentage or mean (SD), except * = median (IQR). ALT: alanine transaminase. AST: aspartate aminotransferase. DBP: diastolic blood pressure. eGFR: estimated glomerular filtrate rate. GGT: γ-glutamyl transferase. HDL-C: high-density lipoprotein cholesterol. LDL-C: low-density lipoprotein cholesterol. SBP: systolic blood pressure.

Number of Patients (Screening)	386
Number of Patients (Included)	317
CANA300 dosage (*n*, %)	
300 mg once daily 150 mg twice daily	144 (45.4) 173 (54.6)
Follow-up time (months) *	20.8 (9.1–35.6)
Patients with follow-up >24 months (%)	43.5
Gender (male/female)	59.6/40.4
Race (%)	
Caucasian	91.5
Latino	2.8%
Asian	0.9%
Black	0.9%
Arab	1.9%
Others	1.9%
Age (years)	62.2 (10.0)
Duration of T2DM (years) *	10.9 (5.8–14.9)
Time of treatment with CANA100 (months) *	9.9 (5.5–20.7)
HbA1c (%)	7.55 (1.08)
Patients with HbA1c > 7%	73.3%
Fasting plasma glucose (mg/dL)	143.9 (38.1)
Weight (kg)	88.6 (18.0)
BMI (kg/m^2^)	32.1 (5.8)
SBP (mmHg)	135.2 (20.0)
DBP (mmHg)	77.2 (11.4)
Heart rate (bpm)	82.4 (27.2)
LDL-C (mg/dL)	81.9 (27.2)
HDL-C (mg/dL)	45.6 (14.0)
Triglycerides (mg/dL) *	138.0 (103.0–201.0)
Uric acid (mg/dL)	5.0 (1.4)
Hematocrit (%)	45.4 (4.8)
AST (U/l) *	21.0 (18.0–27.5)
ALT (U/l) *	22.0 (17.0–31)
GGT (U/l) *	26.0 (18.0–39.0)
eGFR (ml/min/1.73 m2)	85.0 (19.9)
UACR (mg/g Cr) *	10.0 (3.0–33.0)
Hypertension	76.7
Hypercholesterolemia	84.2
Hypertriglyceridemia	41.1
Smoking	
Current smoker	14.2
Ex-smoker	31.5
No smoker	54.3
Sleep apnea	
No	79.4
Yes, without CPAP	9.9
Yes, with CPAP	10.7
Diabetic retinopathy	15.1
Diabetic renal disease	28.1
Diabetic neuropathy	6.9
Coronary artery disease	9.1
Stroke	7.6
Peripheral artery disease	2.5
Arrhythmias	6.0
Heart failure	3.8

**Table 2:** Concomitant antihyperglycemic, antihypertensive, and lipid-lowering drugs in patients switching from CANA100 to CANA300. Data: percentage or mean (SD). ACEis: Angiotensin-converting enzyme inhibitors. ARBs: Angiotensin II receptor blockers.

**Glucose-lowering drugs**	
metformin	87.1
sulphonylureas or glinides	15.1
pioglitazone	1.6
DPP-4 inhibitors	33.1
GLP-1 receptor agonists	47.0
insulin	43.2
insulin therapy (years)	5.6 (2.5–9.8)
insulin dose (U/d)	38.4 (24.3)
basal (%)	73.7
basal-bolus (%)	26.3
**Antihypertensive drugs**	
0 (%)	25.2
1 (%)	26.5
≥2 (%)	48.3
ACEis (%)	35.2
ARBs (%)	37.8
thiazides (%)	27.4
loop diuretics (%)	7.5
**Lipid-lowering drugs**	
0 (%)	18.0
1 (%)	63.9
≥2 (%)	18.1

**Table 3:** Predictive factors of HbA1c reduction during the follow-up after switching from CANA100 to CANA300. Data: mean difference (95% CI). ALT: alanine transaminase. ARBs: angiotensin II receptor blockers. eGFR: estimated glomerular filtrate rate. FPG: fasting plasma glucose. GGT: γ-glutamyl transferase. OD: once daily TD: twice daily. * *p* < 0.05.

Baseline Variable	Mean Change (95% CI) (Unadjusted)	Mean Change (95% CI) (Adjusted)	Mean Change (95% CI) (Best Model)
CANA 300 OD vs. 150 TD	−0.33 (−0.57; −0.10) *	−0.12 (−0.41;0.17)	
Metformin (no vs. yes)	−0.47 (−0.83; −0.12) *	−0.22 (−0.65; 0.22)	−0.36 (−0.66; −0.07) *
ARB (no vs. yes)	−0.23 (−0.48; 0.02)	−0.09 (−0.36; 0.18)	
FPG (per 10 mg/dL)	−0.09 (−0.12; −0.06) *	0.02 (−0.02; 0.07)	
HbA1c (per 1%)	−0.55 (−0.64; −0.46) *	−0.46 (−0.61; −0.31) *	−0.54 (−0.64; −0.45) *
ALT (per 10 U/L)	−0.09 (−0.16; −0.02) *	0.0 (−0.08; 0.07)	
GGT (per 10 U/L)	−0.04 (−0.09; 0.004)	−0.04 (−0.08; 0.01)	
eGFR (per 10 mL/min)	+0.06 (−0.003; 0.12)	+0.08 (0.005; 0.15) *	
Neuropathy (yes vs. no)	−0.45 (−0.91; 0.01)	−0.38 (−0.91; 0.16)	

**Table 4:** Changes in fasting plasma glucose, blood pressure, heart rate, hematocrit, serum lipids, uric acid, serum liver enzymes, estimated glomerular filtrate rate, and the urinary albumin to creatinine ratio in patients switching to CANA300. Data: mean (standard error). * NPT: nonparametric test. ALT: alanine transaminase. AST: aspartate aminotransferase. DBP: diastolic blood pressure. eGFR: estimated glomerular filtrate rate. FPG: fasting plasma glucose. GGT: γ-glutamyl transferase. HDL-C: high-density lipoprotein cholesterol. HR: heart rate. LDL-C: low-density lipoprotein cholesterol. SBP: systolic blood pressure. TGs: triglycerides. UACR: urinary albumin to creatinine ratio.

	Switch to CANA300	Last Visit	Mean Difference (95% CI)	*p*
**FPG (mg/dL** **)**	144.0 (2.2)	129.2 (2.1)	−14.8 (−19.6; −9.9)	<0.0001
**SBP (mmHg)**	135.4 (1.7)	130.1 (1.8)	−5.3 (−8.6; −1.9)	0.002
**DBP (mmHg)**	77.3 (1.0)	74.2 (1.1)	−3.1 (−5.0; −1.3)	0.001
**HR (bpm)**	81.0 (1.7)	81.4 (1.4)	0.4 (−2.5; 3.3)	0.781
**Hematocrit (%)**	45.3 (0.3)	45.5 (0.3)	0.2 (−0.3; 0.8)	0.408
**LDL-C (mg/dL** **)**	82.0 (1.6)	76.7 (1.6)	−5.3 (−8.6; −1.9)	0.002
**HDL-C (mg/dL** **)**	45.8 (0.8)	45.7 (0.8)	−0.1 (−1.3; 1.1)	0.881
**TG (mg/dL** **)**	190.6 (16.3)	183.1 (22.5)	NPT *	0.005
**Uric acid (mg/dL** **)**	5.0 (0.1)	4.9 (0.1)	−0.1 (−0.2; 0.1)	0.541
**AST (U/l)**	25.5 (2.2)	21.2 (1.1)	−4.3 (−8.6; −0.02)	0.049
**ALT (U/l)**	27.1 (1.1)	23.6 (0.7)	−3.5 (−5.4; −1.7)	<0.0001
**GGT (U/l)**	32.5 (1.8)	29.4 (2.2)	−3.1 (−6.6; 0.3)	0.072
**eGFR (ml/min/1.73 m^2^)**	85.0 (1.1)	83.7 (1.1)	−1.3 (−2.8; 0.2)	0.081
**UACR (mg/g)**	52.2 (12.7)	37.8 (9.5)	NPT *	0.007

**Table 5:** Changes in antihyperglycemic, antihypertensive, and lipid-lowering drugs during the follow-up period with CANA300. Data are the percentage or mean (SD). ACEis: angiotensin-converting enzyme inhibitors. ARBs: angiotensin II receptor blockers. DPP-4is: DPP-4 inhibitors. GLP-1ras: GLP-1 receptor agonists.

Drug Class	Switch to CANA300	Last Visit
Metformin (%)	87.1	87.0
Sulphonylureas or glinides (%)	15.1	16.2
Pioglitazone (%)	1.6	6.1
DPP4is (%)	33.1	26.0
GLP-1ras (%)	47.0	63.5
Insulin (%)	43.2	45.1
Insulin dose (U/d)	38.4 (24.3)	35.2 (25.1)
Antihypertensive drugs		
0 (%)	25.2	23.2
1 (%)	26.5	26.7
≥2 (%)	48.3	50.1
ACEis (%)	35.2	33.1
ARBs (%)	37.8	41.0
Thiazides (%)	27.4	26.3
Loop diuretics (%)	7.5	6.6
Lipid-lowering drugs		
0 (%)	18.0	14.0
1 (%)	63.9	60.3
≥2 (%)	18.1	25.7

## Data Availability

Data is unavailable due to privacy and ethical restrictions.
